
JOURNAL INFORMATION
==============================
NLM Title Abbreviation: ScientificWorldJournal
Journal ID: ScientificWorldJournal
NLM Journal ID: 101131163
Journal ID: TSWJ

The Scientific World Journal

ISSN: 2356-6140
EISSN: 1537-744X
Publisher: Wiley

ARTICLE INFORMATION
==============================
PMCID: PMC5917131
PMID: 16547585
DOI: 10.1100/tsw.2006.54
Article ID: 215821
Article version: 1
Subjects: Letter

Carlos Margarit-In Memoriam, 1950-2005

Batlle Daniel
Northwestern University, Feinberg School of Medicine, Chicago, IL, USA
Academic Editor: Daniel Batlle

Publication date: 2006
Electronic publication date: 2006 Mar 14
Volume: 6
First page: 318
Last page: 352
Copyright: Copyright © 2006 Daniel Batlle.
Copyright year: 2006
License: This is an open access article distributed under the Creative Commons Attribution License, which permits unrestricted use, distribution, and reproduction in any medium, provided the original work is properly cited.
License URL: https://creativecommons.org/licenses/by/3.0/

==============================
Letter
*TheScientificWorldJOURNAL* (2006) 6, 318–352
ISSN 1537-744X; DOI 10.1100/tsw.2006.54

TheScientificWorldJOURNAL
www.thescientificworld.com

### Carlos Margarit In Memoriam, 1950-2005

Dedicated by Daniel Batlle
*Northwestern University, Feinberg School of Medicine, Chicago, IL*

Preface by Marina Geli i Fabrega
*Consellera de Salut, Catalonia, Spain*

*Published March 14, 2006*

### Contributors

Michael Abecassis, Northwestern University, School of Medicine, Chicago, IL USA
Montse Amenós, Hospital Vall d'Hebron, Spain
Joaquim Balcells, Hospital Vall d'Hebron, Spain
Pedro Barrios, Hospital General de Hospitalet, Spain
Daniel Batlle, Northwestern University, School of Medicine, Chicago, IL USA
Ronald W. Busuttil, University of California Los Angeles, USA
Ramón Charco, Clinic Hospital of Barcelona, Spain
Roser Deulofeu, Catalan Organization of Transplants, Spain
Marina Geli i Fabrega, Catalonia, Spain
Carlos Ferrandiz, Hospital of Germans Trias i Pujol, Spain
Joan Figueras, University of Barcelona, Spain
Jaime Guardia, Hospital Vall d'Hebron, Spain
Vincent Martinez Ibañez, Hospital Vall d'Hebron, Spain
Eduardo Jaurrieta, Hospital Universitario de Bellvitge, Spain
Josep Lloveras, Universtat Autonoma de Barcelona, Spain
Pau Margarit, Spain
Enric Murio, Consultant Transplant Surgeon, Scotland
Byers W. Shaw Jr., University of Nebraska, USA
Thomas E. Starzl, University of Pittsburgh, USA

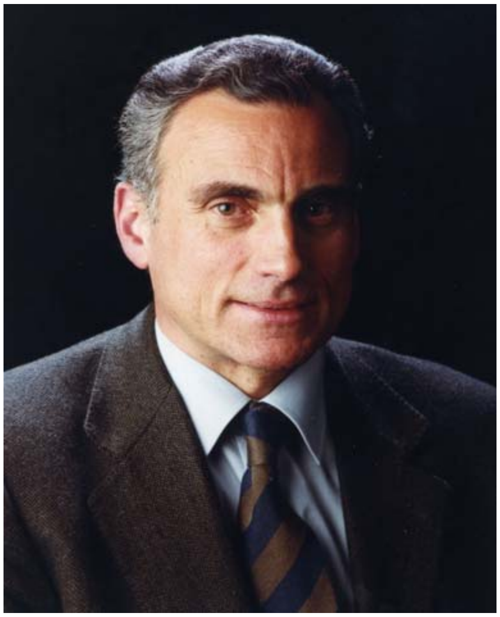

©2006 with author.
Published by TheScientificWorld, Ltd.; www.thescientificworld.com

### TABLE OF CONTENTS

Dedication to Dr. Carlos Margarit Creixell, In Memoriam 1950-2005
*Daniel Batlle* 320
Preface
*Marina Geli i Fabrega* 322
Letter to Ms. Lluisa Colomer
*Thomas E. Starzl* 323
Remembering Carlos Margarit, A Mentor and Mountain Companion
*Ramón Charco* 324
To a Colleague and Friend
*Jaime Guardia* 326
Beyond the First Liver Transplantation in Spain
*Eduardo Jaurrieta* 327
Dr. Carlos Margarit Did Not Have it Easy
*Joan Figueras* 328
See you later, Carlos
*Vincent Martinez Ibañez* 329
Carlos Margarit: A Surgical Symphony
*Enric Murio* 330
Remembering a Surgery Companion and Friend
*Pedro Barrios* 332
Remembering an Exemplary Surgeon
*Joaquim Balcells* 333
Remembering a Consummate Surgeon and Pioneer of Liver Transplantation
*Ronald W. Busuttil* 334
To Carlos Margarit, with Good Memories from Our Days in Pittsburgh
*Byers W. Shaw Jr.* 335
Letter to my friend Carlos Margarit, Pioneer of Liver Transplantation in Spain
*Roser Deulofeu* 335
Remembering Dr. Margarit with Admiration
*Michael Abecassis* 336
Remembering Dr. Margarit and His Impact on The Transplant Community
*Josep Lloveras* 337
Carlos Margarit, The Friend
*Carlos Ferrandiz* 338
Remembering My Boss and Friend
*Montse Amenós* 340
Remembering My Father
*Pau Margarit* 342

Photographs 343

### Dr. Carlos Margarit Creixell, In Memoriam 1950-2005

### Dedication by Dr Daniel Batlle

I want to thank all the contributors to Carlos Margarit In Memoriam, for the wonderful words describing different aspects of his professional and personal life. Further contributions will be considered until the first anniversary of his death. I want to thank *TheScientificWorldJOURNAL* for providing a forum for these tributes in the memory of Professor Carlos Margarit Creixell. It has been very gratifying for me to solicit, edit and, in some cases, even translate into English the testimonies of love and admiration written in his honor. My special thanks to Dr. Marina Geli Consellera de Salut of Catalonia for having written a preface but more important for all the initiatives underway to recognize Dr. Margarit scientific legacy and contributions to Medicine in Catalonia.

Dr. Carlos Margarit Creixell was born on March 2, 1950, in Barcelona. On the morning of December 9, 2005, while skiing with his son, nephew, and a group of close friends in the Spanish Pyrenees, an avalanche took his life. The tragedy was compounded by the knowledge that, Carlos, at 55, was nearing the peak of his medical career. But, the shock wave was felt far beyond Spain. Fellow surgeons were stunned by the sad news, as were his friends in the liver-transplant community.

Carlos had been my dear friend since our days in high school, in the Jesuits of Sarria and, later, in the medical school of the University of Barcelona. Our friendship flourished during the afternoons we would spend together, studying at his home in the Passeig of la Bonanova, as well as our frequent ski trips to Alp, a village in the Spanish Pyrenees.

Carlos was different than most young men of his age, in that he loathed to waste even a minute in trivial pursuits. He was courageous, focussed and driven by a desire to excel in the pursuits that mattered most to him: medicine, family, and sports. Early on, he made it clear to me that he wanted to be a surgeon and make significant contributions to mankind, through medicine. Altruism was a driving force behind the motivation of medical students — more so than it seems today — and no one felt a sense of entitlement or expected that the road to fulfillment would be made easy for us.

Always a high-honors student, Carlos demanded of himself that he be at the top of his game year-round. He used to admonish me by saying, " No se como te lo haces para pasar sin estudiar" which translates into "I do not know how you manage to get good grades, studying so little." While he was saying that in admiration and to be kindly supportive, he would end by saying, "Think what you could accomplish if you were more dedicated." His work ethic and relentless pursuit of excellence inspired me to put more effort into my studies and, ever since, those words have motivated me in my professional pursuits. I moved to the United States after medical school, but the distance between us only served to make our friendship that much more precious.

Shortly after he returned to Barcelona from Pittsburgh, where he trained under Dr. Thomas E. Starzl, Carlos performed Spain's first liver implantation. This was in 1984, and the procedure became a great media event throughout Spain. It took the country's medical establishment by surprise, a reality that did not completely sit well with everyone at the institutions, and indeed he had to face considerable obstacles in his professional career. Admirably, he always took the high road.

Despite the formidable challenges he would continue to face, Carlos enjoyed an extraordinarily productive career. As important, his patients will remember him as a compassionate physician who earned their esteem and admiration .He always will be remembered as a pioneer in liver transplantation and, in my opinion, the difficulties he overcame will earn him not only a place of distinction in our country's medical annals but will make his figure legendary.

By assembling the many tributes from colleagues, collaborators, friends, his son, Pau Margarit, and his assistant of 20 years, Montse Amenos — who both provided me with some of the photos that add visual testimony to the words — my intention was not only to honor Carlos' accomplishments, but also to reflect on what he meant to us as a person outside the hospital. Here, too, he was an extraordinary human being.

His wife, Lluisa has given me permission to quote from his address to guests at their wedding in 1997. I was his best man at his wedding, just like he had been best man at mine, and I was impacted by the sincerity of his words which I believe reflect the essence of his heartfelt beliefs.

> "Our profession often places us next to pain and suffering, and requires an enormous amount of dedication and availability for our patients. Perhaps, for these reasons, we value more the moments of leisure, and we enjoy them to the highest intensity ... like the moments in the mountains, the indescribable sensation of observing the light of Alba illuminating the horizon, while climbing with the skis in the Portillons ... or that July, when, with my son, Pau, we were the first to climb the summit of the Mont Blanc. All those are unique experiences that perhaps only the people in love with the mountains can understand. But, all these passions — my children, my medical career — could not satisfy completely the vacuum that I had because I was lacking being in love.
>
> "For more than a year, now, I am a happy man because I have found the love I was lacking, when I already was thinking it would no longer be possible. I am madly in love. I pray to God that Lluisa and I can enjoy our love for many years to come, and share our happiness with all of you ... family, friends, and colleagues."

It is immensely sad that Carlos was not able to enjoy the fulfillment of his wish for as long as he had hoped. I do find comfort in knowing how happy he had been, and how full of love he was the last time I saw him, only a week before his death. I know how proud of him are Lluisa, Pau, Laura, Ana, Carol, and Eva. Just as his mother, Angeles, his former wife, Merce, his brothers, Felipe, Ricardo, and Jordi, his cousins and many friends, and, just as his father would be.

Not all is lost with his passing, though. Carlos' exemplary life and personal ethics will stand as a source of inspiration for all of us who were fortunate to share his time on Earth. Because he showed us the way, we should now emulate him. His words to me " No se como te lo haces' still sound like music to my ears. Being able to dedicate this tribute to Carlos is very special to me and I know he would enjoy very much reading what we all have to say about him.

Daniel Batlle, MD, FACP
Earle, del Greco, and Levin Professor of Nephrology/Hypertension
Chief, Division of Nephrology/Hypertension
Northwestern University Feinberg School of Medicine
Chicago, Illinois USA

### Preface

By Marina Geli i Fabrega

I am pleased to be able to participate in the homage made through *TheScientificWorldJOURNAL* to Dr. Carles Margarit, a great professional, who unfortunately has abandoned us way to early. Only a few days ago, the Governor of The Generalitat of Catalonia gave Dr. Margarit one of the Trueta medals in recognition of his clinical and investigative work; a medal after his death to recognize his intense activity in both clinical and innovative research in surgery of the liver and biliary tree as well as in the therapy to prevent rejection after liver transplantation.

Carles Margarit was a tireless investigator in the field of liver transplantation and liver-biliary-pancreatic surgery, and he participated in countless projects of research that later became available to the Public Health System for the benefit of all afflicted people.

Thanks to his effort and professional restlessness, he became the pioneer of liver transplantation, having completed the first liver transplantation in Spain in 1984. This was clearly an event of reference, not only for patients from Spain, but also for many countries in Latin America.

Later on, his endless initiative let him develop the first program of Pediatric Liver Transplantation in Spain in the Children's Hospital of Vall d'Hebron. In 20 years of professional dedication to the University Hospital of Vall d'Hebron, he was responsible for 800 liver transplantations and more than 500 liver resections for both primary and metastatic tumors.

Everyone who has known Dr. Margarit will agree with me in speaking highly of him. Not only for his professionality and the important legacy he leaves us for his clinical work, but also for the unquestionable human value of one of the most charismatic and representative figures of Catalonia.

Marina Geli i Fabrega
Consellera de Salut
Catalonia, Spain

### Letter to Ms. Lluisa Colomer

I can't tell you how much I admired your husband, Carlos Margarit, or how deeply I was saddened by the news of his death. Shortly after I came to Pittsburgh in late 1980, Carlos joined our then small group. He and other foreign surgeons who were present between 1981 and 1983 — Bruno Gridelli from Italy, Long Chen from Taiwan, and Bud Shaw, a young American — joined the effort that resembled a crusade. These young men were genuine pioneers. Their respect and affection for each other was immense, and certainly Carlos Margarit was a favored target of admiration.

After he returned to Spain, Carlos kept me in touch with the political maneuvering that slowed his emergence as a premier liver transplanter in Spain and on the world stage. I had the great pleasure of spending the better part of a day with him in Barcelona a few years ago. No one could have been more proud than I was at his remarkable record of accomplishments.

What can one say when such a talent, and more deeply felt, when such a vital humanitarian spirit, is swept away beneath a mantle of snow? It crossed my mind that he may have been taken so young so he would remain that way throughout all eternity. It was a despairing thought, but perhaps it also can bring you some comfort.

Now 80 years old, I am unable to make long journeys, and hope that you can forgive me for not attending the proposed memorial service that may take place in the next few months. Even so, I will be thinking of Carlos on that and every other day, but not solely with sorrow. What I will always remember about Carlos is his warm smile, his courage, and his bold and enterprising personality that left an imprint on everyone with whom he came in contact.

Sincerely,

Thomas E. Starzl, M.D., Ph.D.
Professor of Surgery
University of Pittsburgh School of Medicine,
Thomas E. Starzl Transplantation Institute

### Remembering Carlos Margarit, A Mentor and Mountain Companion

I first knew Carlos Margarit in 1984 when I entered as a resident of General Surgery at the Bellvitge's Hospital. Although then we did not coincide in the same team, our fondness for the mountains and skiing quickly brought us together. Carlos used to run marathons and since he was just a small kid, knew how to ski and sporadically, during the summer months, would climb up the most classic European summits, such as those in the Alps and the Pyrenees. On the contrary, I dedicated myself more to High mountain climbing and when I grew older, also to skiing. In the last few years, I devoted myself to mountain skiing or cross-skiing, which is to ascend to the top of a mountain with skies. When Carlos found out that I practiced that sport, he quickly made plans to start that kind of skiing also. On January 1985, I had planned a winter ascent to the top of the Aneto summit with my ski group. Carlos immediately signed up for it. I mentioned to him that although he was strong and in good shape physically, and even though he was a really good skier, this ascent to the Aneto's in the middle of the winter was considered an ascent for experienced people and it was not recommended for someone that had just started this sport. Obviously, he did not listen to me and went straight out to rent some cross-country skies and he signed up. We started on a Saturday going to Benasque, but we had to leave the car far away from the refuge where we were going to sleep that night. The level of snow was low and the approximation time to the refuge tripled, more than 3 hours. Only with the ascent to the refuge at night and with full moon, Carlos enjoyed it enormously and when he finally arrived really tired, he said to me that he had not had such a good time in a long time, and this was the sport of his life, as it combined cross-country with the surroundings of high mountains and skiing. The next day at 5 o'clock in the morning, Carlos was already up and ready to climb up the Aneto's summit. When we started the ascent, I was sure that he would not make it. Although his pace was very good, the effort that he had to make was much more than mine. He had to compensate the lack of technique with his physical strength. I was astonished to see how someone, who was using High Mountain cross-country skies for the first time, could progress those slopes with such strength and resistance. We remained in Collado of Coronas, just a few meters from the top. He could have reached the top, but he wanted to reserve the strength left in him to enjoy the descent down the glacier. Through the passing of the years, he always remembered that first outing of ours with great fondness.

From then on, I learned to know Carlos Margarit, a tremendously shy person then, introspect, with few but good friends, with a great physical and mental strength. He was a person that transmitted security and was good-natured and tolerant in his outings to mountain skiing. This is the other Carlos, the one that many people did not have the fortune to get to know. During our Bellvitge years, we did a lot of outings to the mountains and shortly after, Pablo, his son, also acquired the taste for this kind of sport.

When in 1989 he told me that if I wanted to work in the Vall d'Hebron's Hospital, I did not have the slightest doubt. It is in this Hospital where he started the Program for the Hepatic Transplants in adults and, together with Dr. Martinez-Ibañez, the Program for paediatrics. A few years later, together with Dr. Astudillo and the team of Pneumology, he started the Lung Transplant. As you can see, he was an entrepreneur and tireless human being.

During the winters, he was always attuned to the weather reports and many times, when it had snowed heavily, we used to escape to the ski tracks. We used to go up and down in the same day. Skiing down the ski tracks is a matter of speech because what Carlos liked the best was to go off the tracks. Many times, we found ourselves in lots of trouble with the "trackers" who wanted to control from where we were descending. I remember one day in the Masella with Carlos and Jonny Birbe. It was a beautiful day with lots of newly fallen snow, and a "tracker" did not allow us to descend the way we wanted. Carlos was very upset with him, using strong language towards the tracker. Perhaps his ways were not always the most appropriate ones, but reason, as usually, he did not lack. Dr. Carlos Margarit did not like anybody to tell him what to do; he did not like to be bossed around. The same thing went for his hospital life and more so when those that tried to boss him around did not have, in his opinion, the professional level they should have.

Also, on the mountain he was a persistent person, even stubborn, I would say. On the days of adverse weather reports, he wanted to go out. Sometimes, like the day when we climbed to the summit of the Maladeta, he wanted to continue although the weather was bad. Truly, we had a really bad experience then. Carlos was a risk-taker and that is a quality that differentiates people who are winners from those who are not.

Daily work with Dr. Margarit was not easy. He was a hard-working and persistent person who did not relax, not even for a minute. When he chose a goal, he did not cease until he got it. It is true that sometimes he crashed with impassable walls, but nobody ever would deny that he always held his head really high. When we went to the mountains and we had to arrive at such or such place, to a refuge or whatever place, he did not stop until we reached it. One Sunday night, he called me on my cell phone to explain to me that he reached the top of the summit of Possets by Viadós. This classic ascent is considered to have the longest descent with skies of the Pyrenees. He told me that in the middle of the way going up, one of the straps of his skies broke and he had to go up walking until the top, sinking in the snow up to his knees. According to him, the descent walking was exhausting. Anybody that had a problem like that on the way up usually just goes back and returns to his or her house. But not him, he did not stop until reaching the top.

Finally, I just want to emphasize Dr. Carlos Margarit's honesty and his altruistic facet. It is true that Dr. Margarit did not give anything away, but in my personal opinion, he put the ways so everyone would find his or her place. After being so many years with him, the possibility of going to work to another Hospital was offered to me. In fact, Carlos, as always, was ahead of the events. One day he told me that there was a place open in the Hepatic Transplant team at the Clinic Hospital of Barcelona and because of my personal characteristics, my professional formation, and my good relation with the surgeons of that Hospital, they would offer the position to me. I told him that if that was the case, he would be the first to know. Just few weeks after this, they asked me to work with them. At the beginning, he tried to convince me not to go. A few days later, when I told him what the change meant to me, he told me: "Although I will regret it, in your shoes, I would not have the slightest doubt." He was always proud that a surgeon that he trained in Transplants would get to work at the most prestigious Hospital of Spain and one of the most important in Europe in the field of Hepathology. The same way he was proud that Dr. Murio or Dr. Hidalgo, two transplant surgeons also trained by him, hold today leadership positions in Transplant teams in the United Kingdom.

I believe that Carlos Margarit can rest in peace knowing that we have to give thanks for everything that he gave us. His head was full of goals and projects on every level, one of which was doing the marathon in less than 3 hours and another was to start an Intestinal Transplant Unit. The first one cannot be done anymore, but the second is in the hands of the Hospital where he worked more than 20 years.

Ramón Charco, MD, PhD
Consultant Liver Transplantation Unit
IMDiM, Clinic Hospital of Barcelona

### To A Colleague and Friend

On the morning of December 9th, 2005, an avalanche of freshly fallen snow took the life of Carlos Margarit while he was practicing his favorite sporting activity in the Spanish Pyrenees. Everyone who knew Carlos, deeply shocked by the news of his death, will maintain the memory of his person and life as an example to follow.

His death is a significant blow to the field of Medicine in our country. A surgeon with capital letters, of those that there are very few left, he had a complete dominance of surgical techniques and, at the same time, had exceptional diagnostic skills and knowledge of medical treatments.

A person with a mind that never stopped, always looking to improve himself, he went to Pittsburgh shortly after he finished his surgical residency at the Hospital Universitari de Vall d'Hebron to learn liver transplantation under Dr. Thomas Starzl, the pioneer of this procedure. After he returned to Barcelona, at only 34 years of age, he performed the first liver transplant in Spain, together with Dr. Eduardo Jaurrieta, in February of 1984 in the Hospital de Bellvitge. In 1986, he transferred to the Hospital Vall d'Hebron, also in Barcelona, and organized a group of very active surgeons and his team initiated the liver transplantation program in our Hospital. The recipients of more than 900 liver transplants are witnesses of this dedicated professional life and the human quality of Carlos and of everyone in his group of collaborators; many of them now lead units of surgery and liver transplantation in hospitals in Spain and Europe.

Carlos not only knew how to innovate different techniques in the field of surgery and transplantation, but also to lead numerous projects of research in the field of immunosuppression and rejection.

The most remarkable characteristic of Carlos' personality was, without a doubt, his desire to learn and to teach, combined with his capacity to work tirelessly with personal dedication, coupled with a nonconformist attitude when dealing with the issues that hospitals face on a daily basis. A serious person, somewhat shy, with a generosity without limits demonstrated not only with his patients, but also with his collaborators, from whom he demanded a lot, but to whom he was capable of giving much more.

Spanish surgery has lost one of its greatest figures, one who still had a brilliant future ahead of him. The many disciples he has left, well prepared and with a capacity to lead, will continue his work to maintain his legacy. Those of us who worked with him for the last 20 years will remember Carlos everyday, and his memory will help us to continue his efforts together with the efficient collaborators who are continuing his exemplary work.

Dr. Jaime Guardia
Jefe de Servicio de Medicina Interna-Hepatologia
Hospital Universitari Vall d’Hebron
Universidad Autonoma de Barcelona
Spain

### Beyond the First Liver Transplantation in Spain

In the summer of 1983, Carlos Margarit and I came back from our stage in Pittsburgh and Hanover, respectively. Both of us had been abroad to learn liver surgery and liver transplantation.

Despite that we were working in different departments of Surgery in the same hospital and we were surgeons of 33 and 35 years old, we decided to work together. So, we prepared the protocols and encouraged some clinicians from other specialities and nurses to create a group motivated to do the first liver transplantation in our country. At that time, very few people thought that this was possible because only four groups in the world had an active program in liver transplantation.

Finally, at the end of 1983, we were ready to transplant a liver. The opportunity with a patient with a good indication and the donor arrived in mid February 1984. As we had planned, we went together to extract the donor's liver. After that, I did the hepatectomy in the patient and Carlos helped me, and then he implanted the liver and I helped him. The result was good and the impact in the hospital and in the society was unbelievable. However, this was not an isolated fact. In 6 months, we transplanted 10 livers more and 7 of the patients could go back home. People who knew results in the early 1980s from other groups could appreciate that our results were good.

For me, it was a great honour to work with Carlos Margarit in that project. He was a very hard worker, and a very good clinician and technician. He was, until his death in an accident in December 2005, a model for young surgeons for his abilities and dedication to liver and transplant surgery. For Catalan and Spanish surgery, there is now a gap that cannot be replaced.

I will never forget the time I worked with Carlos Margarit. Our work at that time was hard, but also very gratifying.

Eduardo Jaurrieta
Professor and Chairman in Surgery
Hospital Universitario de Bellvitge
Barcelona, Spain

### Dr. Carlos Margarit Did Not Have It Easy

I consider that Carlos Margarit had a difficult life. For many surgeons of our age, Carlos was a mirror through which we could measure our progress. Dr. Margarit's merits have been widely praised in the press by influential personalities in the world of surgery and the university. He was the first to perform Liver Transplantation in Spain. He had one of the best Curriculum Vitae in the world. He was both an academic surgeon and a model researcher, both as an oncologist and as a liver surgeon. He was also an irreplaceable figure as head of the Liver Surgery and Transplant Unit for Adults and Children at the *Vall d'Hebron* Hospital.

What is probably not so widely known is that he was obliged to leave the *Bellvitge* Hospital, where he performed the first liver transplant in Spain, because he was removed from the Transplant Program; that he was obliged on two occasions in the middle of his career to enter competitive examinations before being awarded the position of Professor Lecturer at the *Unversitat Autonoma de Barcelona*. It is also not generally known that, in spite of his experience and capacity, which many would now characterize as irreplaceable, his Liver Transplant Unit in the Hospital was on the point of closing as a result of the intrigues of surgical politics — the lawyers who were handling the case can bear witness to this aspect. Perhaps the most unprecedented occurrence was that only a few months ago, he did not even pass the first round of the selection process for the position of Senior Professor of Surgery. The Spanish University must be in a pretty poor state if it was unable to recognize the academic merits of Carlos Margarit. However, the strangest thing is that those who strewed the most obstacles in the path of Carlos Margarit during his life are now the first to jump on the mediatic bandwagon surrounding his death.

Joan Figueras, MD, PhD.
Professor of Surgery
University of Barcelona, Spain

### See you later, Carlos

I went to meet him at a conference centre in Barcelona where he was giving a talk on his experiences in Pittsburgh. I had been in touch with Dr. Marc Rowe, my American mentor in paediatric surgery who had just arrived in Pittsburgh in 1983 and with whom I had worked and built up a close friendship in Miami, to arrange a stay with Dr. Thomas Starzl in Pittsburgh.

During his talk, Carlos was serious, contained, but hopeful. After the lecture, I contacted him and was soon able to attend the new liver transplantation sessions being held in his hospital. I had the privilege of attending the first adult liver transplant in February 1984. Later that year, I went to Pittsburgh and my professional life changed in attitude, seriousness, and demand. When I returned to Barcelona, Carlos and I noticed that we shared goals and philosophy. He was a general surgeon with the delicacy of the best paediatric surgeons — decided, decisive, and sure in his surgical gesture and criteria. His doubts evanesced into himself, into his shyness. Only on very few occasions would you see him in a dilemma. Whenever he had a difference of opinion in a place where he felt ill at ease, he would go tense, but when he did eventually decide to speak up, his professional arguments invaded the room. Carlos was brilliant at presentation; clear in his message; straightforward, but deep.

Our friendship was established quickly. Hours of conversation day and night, silences, and waiting for calls paved the early days of the first paediatric liver transplantation programme in Spain, in 1985. We had some organisational differences, but almost never about clinical criteria and, in the worst moments, safeguarded our friendship and our mutual respect.

In April 2001, when I became chairman of Paediatric Surgery at my centre, Carlos was one of the first to congratulate me and offer his support. From then on, our lives diverged professionally, but with complementary aims. We helped each other. Our conversations could be serious, talking about transplantation or personal matters in my office, or shorter when we met in the labyrinthic corridors of our children’s hospital.

Carlos was always daring in his actions. He always assumed the risks, but suffered, particularly for his patients. The example I always keep with me and for me, and share with young surgeons is: Although a surgeon has to be a good researcher, and Carlos was, and a good communicator, and Carlos was brilliant, the most significant thing is to care for and about patients, and Carlos did. This was his root. He was always motivated to improve. A great surgeon, enormous.

Carlos left us in an accident in a place he loved, in the mountains surrounded by snow. Good sportsman, marathon man, cyclist, experienced skier. The broken snow robbed us of his presence in our daily routine. Today his friends, still in shock and angry, remain incredulous.

His example will stay in my memory forever.

Our sincerest thanks, Carlos, for all you have done for surgery.

See you later, Carlos

Vincent Martinez Ibañez
Chairman of Surgery
Hospital Universitari Vall d’Hebron
Barcelona, Spain

### Carlos Margarit: A Surgical Symphony

Daybreak on October 31st of 1988 marked a milestone in the history of General Hospital Vall d'Hebron as a Surgical Center. On the morning of the day before, Dr. Carlos Margarit succeeded in a competitive examination that confirmed him as an exceptional surgeon and Chief Director of the Surgery Services at that Hospital. By mid-day, a team for hepatic transplants was established there.

I still remember with moving emotion when, in one of its corridors, Dr. Jose Bonnin, Chief of Surgical Services, offered me to participate in that program. That was something that I did not expect; nonetheless, I agreed immediately and without any doubt in my mind. On that afternoon, we flew to Valladolid where the family of a young boy who had just died in a car accident agreed to a multiorgan donation. At that time, I was recently qualified as a surgeon and without any experience in transplants, but this fact did not at all worry Dr. Carlos Margarit. This surgical operation was my first contact with him in the operating theater and I knew then that his influence would change my professional life forever.

The first year and a half was very hard, not only because Carlos was a very demanding and rigorous person, but also because of all the difficulties of starting a new program so ambitious in a Hospital that sometimes could not keep up the pace.

I still do not know from where Dr. Carlos Margarit took the strength and the determination to push us all in a new and different field, to work more and better. He was always the first to give an example of dedication, capacity of sacrifice, generosity, and resilience. His strength came from a blind faith in what he was doing. He had an informed opinion on almost everything and he did not give those opinions away freely, but he would defend them with passion if that was the case. He was a person with solid principles, fully convinced of his duty. I do not think he knew that he inhabited a very high summit inaccessible for most of us, at least for me. I did not share his fondness for the mountains and because of this, we did not have many leisure moments together. Therefore, the best way for me to know him was through his work. I am sure that Ramón Charco, a dear friend of Dr. Carlos Margarit and an outings partner, can give better evidence of his vitality and of his personal warmth in a more relaxed atmosphere.

I spent countless mornings, evenings, and nights with Carlos for more than 15 years, head to head, with our eyes and hands fixated on the surgical field. During the first months, it was an intimidating experience. He prepared his most complicated interventions a long time ahead and I am sure that he saw each possible surgical field before each intervention. The morning before a complicated case, as most of them were, he was the first to arrive to his office, ready to enter into the operating theater. Dr. Carlos Margarit would greet his patient first; he smiled and gave him confidence, the kind of confidence that only someone that assumes great responsibilities and risks can offer.

Dr. Margarit's technique was one of exceptional magnitude. It was based in the deep knowledge of anatomy and a great honesty that led him to achieve the highest quality in his work that obviously later on derived the most extraordinary results. His ability to catch hold of oncological roots was impeccable. For this, he never sacrificed effort or time, nor did he avoid any risks, until he reached the objectives of excellence that he wanted. Dr. Carlos Margarit was very thorough in every detail. He put the patient on the table, adjusted the lighting himself, and personally prepared the operating field with mathematic precision. He was not always predictable because sometimes he liked to probe different techniques, all personalized for each case and patient. Dr. Margarit's mind wondered without ever resting. In the most delicate situations, his genius would surface — through improvisation, courage, and steadfastness — to regain control of any given situation. He treated tissues with astonishing delicacy and care, but at the same time, he was energetic, quick, and decisive. He was not whimsical with instruments, but he was very demanding and precise. To instrument him was always a challenge of ability and anticipation for his assistants. With time, I learned how to read his thoughts, to go along with his gestures and movements, and to enjoy immensely with what he knew to do best, which was nothing less than a real Surgical Symphony. His self-discipline would keep him watching even for the most insignificant details and very often he would stay until the last skin stitch was done. Everything had a reason and a way of doing it, and that was the way it should be done. I am sure that outside the operating theater he was also that same way and more. Carlos was a polifacetic person, who poured his personality on everything he did. He was long-distance runner of incorruptible principles who, more than once, pulled me and others through with the tenderness of a severe father. Dr. Carlos Margarit lived life with the intensity and with the courage of one who knows about the privilege and the duty of giving the best of oneself with generosity.

Now that Carlos is gone, I want to believe that my hands move as they learned from his, that my mind now uncoupled and orphaned, can apply a surgical logic the same way he did, and that my personal discipline and professional honesty will continue to be inspired by the memories of that part of my life that I shared with him.

Enric Murio
Consultant Transplant Surgeon
Western Infirmary
Glasgow, Scotland

### Remembering a Surgery Companion and Friend

It is not easy to talk in the past tense about a person when you can feel their presence still. Several months have passed since the accidental death of Carlos Margarit and it is still difficult for me to assume this fatal event. In our own lives, there is always a person whom we believe will never die, and that something of them will survive forever in ourselves.

I will briefly recall some aspects of Carlos' personality that I will always keep in my memories, and those qualities that made me better at a certain point in time, and from now on, and more than ever, will become part of my personal and professional background.

Carlos was my first surgical attending when I was in training as a surgical resident. It was not a chronological issue, but the fact that makes real sense to the relationship between the trainer and the trainee. In a highly professional environment where the dexterities and surgical skills already are the highlight of surgical training, Carlos was able to add other values like scientific methodology. Long night calls shared with him were not only limited to waiting for complex surgical cases, but also, to a large extent, to engage in surgical discussions of clinical cases, reviews of surgical reports, or simply, difficult decision making based on a single X-ray. The partnership of that young surgical team on call, along with the desire to improve in knowledge and expertise, led us to claim, several times, the transfer of patients from other facilities to our hospital. So many years and so many night calls shared together built up a strong feeling between us and our dedication to surgery became some sort of an obsession. Even at off-duty times, we used to share our hobbies for motorbikes, certainly as an excuse so that we could keep talking about medicine.

During the postoperative period and follow-up of these patients, Carlos was enormously critical, applying the rule to himself that when it comes to morbidity, unless proven otherwise, it should be blamed on the surgical procedure, and therefore, the surgeon's responsibility. At a time with no computers and computerized databases, he used to record his own data in a notebook that he always had with him. Being conscious of his structured personality, all these notes must be still in some case box, waiting for somebody to go through his difficult handwriting.

In the operating room, Carlos was a highly skilled surgeon among different surgical fields, but what really made a difference was his extreme meticulousness and patience when undertaking difficult surgical decisions. He used to say that patients never die during resection time, but later, due to complications, during reconstructive procedures. It was his own way of showing respect for his patients, avoiding complications, and betting, at the time of surgical procedure, for the better outcome.

Carlos' research activity at that time of his surgical career was a pending subject; the daily workload was a priority. One of the aims, along with a great attraction towards medical development in the USA, was to apply for a grant in surgical oncology training at the Memorial Sloan-Kettering Cancer Center in New York. But, once again, and after several months in that city, his analytical and pragmatic mind pushed him to move to Pittsburgh where Professor Starlz was performing several liver transplantations every week. He realized then that a good training in this surgical field could be applied on returning to Barcelona. In Pittsburgh and during prior residency training, we also shared great times and encouraging career projects for the future with Jordi Colomer and Carlos' family.

In Spain, we all know Carlos' professional achievements. He could reach most of his goals. He was a pioneer, a day-in and day-out surgeon, and an innovative in the field of organ transplantation and other surgical specialties, besides a great friend.

But, all of us who got to know and love him also know he could do so much more. Unfortunately, surgery lost time to advance the ability and professional capability of Carlos Margarit. For his friends and family, what really matters is that we lost him when he was needed the most.

See you forever, master and friend, Carlos.

Pedro Barrios MD, PhD
Head of General Surgery and Oncology Department
Hospital General de Hospitalet, Barcelona, Spain

### Remembering an Exemplary Surgeon

While writing these words, I was looking at the peak of the Pyrenees where, one Friday morning, my colleague Carlos Margarit died doing one of the three things he loved most: family, liver surgery, and skiing.

A snow avalanche surprised him while he was skiing with his son and other friends. Everybody except Carlos survived. I heard about this tragedy the same evening and was astonished at first by the incredible news and felt very sad for a long time.

He was my boss, teacher, friend, and colleague for many years. The first time I met him was in the operating theatre when I was a medical student finishing my third year. A few months later, he went to the U.S. for a year. He went first to Sloan Kattering Memorial Hospital in New York and later to Pittsburgh with Dr. T. Starzl, learning about liver transplantation. After his return to Barcelona, he and Dr. Eduardo Jaurrieta performed the first liver transplant in Spain in 1984. Carlos Margarit transferred to the Hospital Vall d'Hebron when I was in my fifth year of surgical residency and we met again. He was then forming a liver transplant team and he invited me to join. A new period began at our hospital in 1988 with our first liver transplant. Since then, we have performed approximately 800 adult and pediatric liver transplants. It has been an exciting period that all the members of the team, not only the surgeons, have enjoyed. All the members of the team remember Carlos for his enthusiasm and courage and for going beyond the frontiers of surgery.

Dr. Margarit was not only an exceptional surgeon, but also a good clinician, a tireless researcher, and an innovator. Patients and their families admired him and many of them were at his funeral, honoring his memory with their presence and giving their last thanks for all he did for them.

All we Spanish surgeons have lost our liver transplant pioneer. He was an exceptional figure for all of us and the mirror in which new young generations of surgeons should look and take example. For me, he was like my older brother and I feel like an orphan, but I must continue his efforts and make his dreams for liver surgery and plans for a new intestinal transplant program to come true. He is sorely missed and I will never forget him.

Dr. Joaquim Balcells
Chief, HBP Surgery and Liver Transplantation Unit
Department of Surgery
Hospital Vall d'Hebron
08035 Barcelona, Spain

### Remembering a Consummate Surgeon and Pioneer of Liver Transplantation

Carlos Margarit, MD, PhD, an internationally known liver transplant and hepatobiliary surgeon who was the pioneer of liver transplantation in Spain, died on January 9, 2006 when he was trapped by a snow avalanche while skiing with his family. He was 55.

Dr. Margarit received his medical degree in 1973 from Universidad de Barcelona and completed surgical residency at the Hospital General Vall Hebrón, Barcelona. In 1982, he served as a fellow in hepatobiliary surgery at the Memorial Sloan-Kettering Cancer Center, New York, where he studied under the renowned liver surgeon, Dr. Joseph Fortner. Following his training in New York, he was accepted in 1983 for transplant fellowship training at the University of Pittsburgh and studied with Dr. Thomas Starzl. That is where I met Carlos, and immediately I recognized his outstanding abilities as a surgeon who could quickly and accurately analyze the most difficult surgical problem and expertly effect a surgical remedy. His keen intellect, love for his work, and warm and engaging personality endeared him to colleagues and patients alike.

After completing his fellowship at the University of Pittsburgh, he returned to Spain, where he was appointed Director of the Liver Transplant Program at Hospital de Bellvitge, Barcelona. There on February 23, 1984, he performed the first liver transplant in Spain. In 1988, he became Director of Liver Transplantation at the Hospital Vall Hebrón, Barcelona. He served in this post until his death and performed nearly 700 adult and 200 pediatric liver transplants during his career.

Dr. Margarit was the model academic surgeon who was a true “triple threat”. He was a superb clinical surgeon, actively and effectively engaged in clinical and basic research, with a genuine love for teaching students, residents, and fellows.

Throughout his career, Dr. Margarit investigated and contributed to many aspects of liver transplantation, and that work has improved the lives of countless patients. His areas of concentration included development of improved strategies for immunosuppression, management of hepatocellular carcinoma, and protection against ischemia-reperfusion injury in the liver allograft.

Dr. Margarit was not only a consummate surgeon, but also a devoted family man. His legacy will live on among his five children, his wife Lluisa, his family, and his patients. All of us who considered him a close colleague and friend will mourn this tragic loss, but perhaps can find some solace in knowing that we are better people just by having been associated with Carlos.

I will miss him very much.

Ronald W. Busuttil, MD, PhD
Professor and Executive Chairman
UCLA, Department of Surgery

### To Carlos Margarit, with Good Memories From Our Days in Pittsburgh

My fondest memory of Carlos involves a party I hosted at my house in Pittsburgh. It was in the summer of 1983 and we had been working very hard for Dr. Starzl for weeks without rest. In fact, the original party was supposed to have occurred the day before, but a transplant forced us to postpone it a day or two. After a wonderful evening of drinks and food, we spent the rest of the night singing Italian, Chinese, Japanese, and, of course, Spanish songs on my back porch. I can still see Carlos singing his heart out, faking the words in those languages none of us understood, but happy, truly happy.

Carlos was a great friend with whom I lost contact more than a decade ago. A tribute to his powerful personality, I remember him as though those 10 years were only yesterday. We will all miss him terribly.

Byers W. Shaw Jr., M.D.
Professor and Chairman, Department of Surgery
College of Medicine, University of Nebraska
Omaha NE

### Letter to my friend Carlos Margarit, Pioneer of Liver Transplantation in Spain

It is not only in my name as your personal friend that I am writing you this letter, but in the name of the whole transplant community, as it was you and your team that pioneered liver transplants in the Spanish state. You did this in Barcelona that 23rd of February 1984.

Not only did you bring your surgical techniques in transplant, gained in Pittsburgh, you were also able to transmit and demand organizing skills from those of us who had this responsibility at that moment in time. These skills were adapted to construct the first Catalan transplant model, transmitted to communities throughout the rest of the Spanish state and from there to the rest of the world, with highly successful results.

Your air, perhaps a little shy in that year of 1984, which you have conserved until now, in your maturity, was surprising, contrasting with your professional dedication which launched you ahead, with all your knowledge and skills, drawing your team into the most difficult surgical cases, the most complicated transplants, surgical commitments and research at the highest level.

Personally and professionally daring, a surgeon of firm, sure hands, with skills and ability so extraordinary that, according to your closest colleagues, you finished your operations with "clean gloves".

You have been seriously committed to your work and your patients and have closely involved your collaborators and disciples with the energy and example of someone who, not even when young, allowed him any weakness. Someone who has tried and overcome successfully all difficulties, and who made the most complicated medical situations look easy, up to the day of his death.

Many times against the wind, misunderstood and opposed by others you maintained your honesty in thought, medical criteria and decisions. You always put the needs of your patients first, which made you truly unmovable when it came to defending their interests. You have died as you lived, fighting the elements, in this case the snow; in the mountains you loved so much.

The "death" you have stolen from so many lives has taken you today......, but will never be able to remove your memory from our hearts. Nor the projection of your professional work in the world of medicine, as you will always be with us.

Rest in peace Carlos... dear friend

Dr. Roser Deulofeu
Director Catalan Organization of Transplants
Barcelona, Spain

### Remembering Dr. Margarit with Admiration

Dr. Margarit performed the first liver transplant in Spain in 1984 shortly after the introduction of Cyclosporine made liver transplantation a viable therapeutic modality for patients with end-stage liver disease. Dr. Margarit embodied surgical innovation and the surgical work ethic. I had the honor of meeting Dr. Margarit at various international scientific meetings and was always impressed with his charm and intellect.

The sudden nature of his death left his friends and colleagues numb with shock and disbelief. As we have had an opportunity to reflect on his life and death, there are clear parallels between his surgical career and his sporting activities. Both required a high level of skills and courage, and a continued desire to improve the status quo.

Dr. Margarit will be remembered by all with fondness and admiration.

Michael Abecassis MD MBA
J. Roscoe Miller Distinguished Professor
Departments of Surgery and Microbiology/Immunology
Feinberg Northwestern School of Medicine
Chief, Division of Transplantation
Northwestern Memorial Hospital
USA

### Remembering Dr. Margarit and His Impact on The Transplant Community

It was during the afternoon of December 10, 2005 when we heard the terrible news that Dr. Carles Margarit died, buried by an avalanche while skiing in the Pyrenees.

Carles Margarit was Professor of Surgery and Chief of the Hepato-Bilio-Pancreatic Unit and Chief of the Adult and Pediatric Hepatic Transplant Program of the Vall d'Hebron Autonomous University Hospital of Barcelona. Born in 1950, son of a renowned thoracic surgeon, he earned his Medical Degree in 1973 at the Barcelona School of Medicine, his Doctorate in 1986, and Professor of Surgery in 1995. In 1982–83, he went to the USA for training with Prof. J. Fortner at the Memorial Sloan Kettering Cancer Center of New York and with Prof. Thomas Starzl at the University of Pittsburgh.

In February 1984, he performed with Dr. E. Jaurrieta the first liver transplant in Spain and organized the first Liver Transplant Program. He also pioneered the first Pediatric Liver Transplant Program that started in 1985. Carles Margarit performed over 800 liver transplants in adults and children and was the introducer of graft reduction, split liver in children in Spain. Over the last several years, Spain has been leading liver transplantation activity internationally, with more than 1000 transplants per year (over 24 LTx pmp in 2004) and Prof. C.Margarit had much to do with this success, through his activity and the numerous surgeons that were formed in his team.

In spite of the fact that he was basically dedicated to clinical activity, Carles Margarit was passionate about experimental surgery and headed many research programs. Other research fields of his interest were in immunosuppression, tolerance, transplantation immunology, liver preservation, and hepatic neoplasias. He published more than 160 articles in scientific journals of internationally acknowledged prestige and contributed more than 700 presentations to national and international meetings.

He was an excellent clinician, a tireless worker, and an ambitious innovator, and behind a certain shy character, there was his tenacity, conviction, and generosity that pushed his collaborators to always go further.

During the last 4 years, he served two terms as President of the Catalan Transplantation Society and the Catalan Transplant Foundation with great dedication and efficacy, providing increased prestige to both institutions.

He loved sports and practiced mountaineering, skiing, and running regularly, which he used to say constituted important help for his professional work. His personal record as a marathon runner was in 1991 in New York.

Carles loved and had a passion for his family. With Lluïsa, his wife, he had three daughters who he adored, and with his two older children, he shared the passion for the mountain.

Carles Margarit has now become part of the medical history of our country and we also have to say with great sorrow that the transplant community has lost an invaluable asset.

Josep Lloveras
Universtat Autonoma de Barcelona
Spain

### Carlos Margarit, The Friend

A few months ago, our beloved and dear friend, Dr. Carlos Margarit Creixell left us for good. I am being invited to write about my friendship with him. His broad and recognized professional merits will be widely collected by other professionals that worked closely with him. I just want to write about his personality.

I think that if I had to define Carlos' character, I would sum it up by saying that he was a tireless worker with an indomitable spirit striving for continuous excellence. I would also say that behind his shy and reserved demeanor, a man with an enormous heart was hiding. I would say that through his studies, hard work, and dedication to Medicine, he did a lot, much more than asked for, for his patients. There is no doubt that for him Medicine existed first and then all the rest.

As in his life as a Scientist, Carlos was also, from the personal point of view, a leader. A leader not in the classic sense of driving the masses; this was never his style. He was a rather humble person, introspect, with an unbelievably high level of self-demand that was out of the ordinary. What he demanded from himself at the professional level he demanded also in his personal life. He had to be always the best. All that he did, he did it right, to the highest level. He never did anything just to gain praise or recognition from others. He would do it because he demanded it from himself.

Two of Dr. Carlos Margarit's most distinctive traits were his passion for sports and nature. This too made our friendship stronger, beyond Medicine. He was a tireless worker, but he always found time to dedicate a part of his unlimited energy to both passions.

If a day only had 24 hours, there was no problem. He would find the 25th hour to go jogging, even if it was at dawn, no matter where he was at the time. It could be Barcelona, New York (where he even got to run the Marathon), Tokyo, or Cape Town. His running shoes were always part of his luggage. During the winter weekends, he would also find a moment to go skiing whenever he had the chance, but he would not do it in the regular tracks at ski resorts where most of the normal people do it. No, that was not his style. He had to look for a challenge… like climbing to the highest peak wherever he would be, carrying his skis on his shoulders, and then descending slopes that probably no one, or just a few people like him, had skied before. His life was a continuous challenge for improvement. Unfortunately, in a twist of fate, he left us forever on one of those rides.

Carlos was a nature lover, one of his best girlfriends, the one that he liked to challenge with great goals.

I often remember traveling together with both of our families. On those trips, he would always manifest his passion for new achievements. Achievements that he kept for himself for his personal satisfaction and that he never bragged about.

In particular, I remember fondly two of our journeys together; one of them was to climb the highest mountain in Europe, the Mont Blanc. For this purpose, we went to Chamonix and remained there while waiting for the weather reports that would allow us to climb. We were there for a few long days, after which — even though it was pouring rain on that day and the weather reports were all but good — we climbed to the first position, the Refuge. We passed the night there and waited until the next morning for the weather to calm down a little and then we would continue to climb to the top of the Mont Blanc. There again, he showed his character. At one in the morning, the scheduled time to start the conquest of the summit, the weather reports were still very unfavorable with very high winds. Any other mountain climber, including myself, would have given up; not him. He just said: "Let's wait a few hours more to see if the wind dies down and see if we could try it later." By seven o'clock in the morning, the conditions remained the same. I remember vividly what he told me: "Carlos either we climb up now or we have to wait until next year." Faced with that choice, my vote was in favor of letting go. Carlos, finding it difficult to renounce his goals when faced with any given challenge, said: "Let's try it, we could always return if we see that we cannot make it!" From that moment on, I knew that he would succeed and he did. Thus, we started to climb until a few meters from the summit, facing an atrocious and persistent wind that made it difficult even to stand still. I had to give up. He did not. He continued and, naturally, reached the top just at the same moment I returned to the Refuge and waited for his return. I believe that Dr. Carlos Margarit was the only person on that day to reach the top.

This was Carlos, a courageous, decisive human being without any boundaries for new challenges and fearless to take new risks. In his professional life, he was the same. He conquered all he reached for, but not always in an easy manner, as many of the writings now appear to show. It was not like that. In his professional life, he had to fight against all odds. He spent many hours of dedication and fighting continuously for inaccessible achievements for most mortals. Carlos knew no limits. He was the same on the mountain or wherever he went. It had to be done and he did it. Even though difficulties were multiple and extreme, he never minded that. For Dr. Carlos Magarit, there were no limits to what a human being could do. There, at almost 5,000 meters high, he showed his winning character once more, fearless of any difficulties along the way to excel himself.

Another journey that I keep fond memories of is our adventure in the United States of America in its Northwestern National Parks. I went there with our respective families one August to take advantage of the annual Convention for Organ Transplants that was held in San Francisco and Carlos was already there. Once again, he demonstrated his untamable character. We visited all the Parks there were and, in each, we had to do something “extra”. It was not good enough to visit them as other people did. He had to do something more. For instance, if it was advised that 2 days were needed for the descent into the Colorado Canyon, we had to do it in one because the next day we had to go somewhere else. In these circumstances, his normally serious aspect was transformed into a personal satisfaction and a contented smile. He loved nature and he lived for it; he always kept it inside his mind.

That was our Carlos — a hard worker, a sports and nature lover, with a tremendous thirst for seeing and knowing all without any boundaries, with a level of self-demand out of the ordinary.

Dear and beloved Carlos, your friends will always have you among us.

Rest in Peace.

Carlos Ferrandiz
Professor, Autonomous University of Barcelona
Chief of Dermatology
University Hospital of Germans Trias i Pujol
Barcelona, Spain

### Remembering My Boss and Friend

Neither his strength, nor his intelligence, nor his youth could help Carles to escape his death; he had a leader's death. The mountain he loved so much claimed him, but it could only keep his body, his spirit will always be with us.

For the last 20 years, my professional life was spent close to Dr. Carles Margarit, from the first transplant (Berta's transplant in 1985), when he came to ask me to search for some documents of the patient he needed, until the last time I saw him on the 7th December 2005, when he locked his office, held his suitcase with his left hand, and with his right hand grabbed my arm and told me, "Montse, I've got my mobile, we'll talk about bowel transplants next Monday." I think he did not want the thread he weaved around him to work loose. He had years of misfortunes, fights, obstacles, hard times, but at the same time, he was full of life, gratitude, positivity, and also good times.

Briefly, I will explain my memories of my time spent working with Dr. Margarit during those years and his most human side.

I remember in 1988, when we started the liver transplants in adults and with great difficulty built the Liver Transplant Unit, allocated in the old dining room on the 4th floor of The General Hospital because at that time they didn't use it as a canteen. We were able to make good use of a little space of 6 × 6 meters, similar to a square. We put up three walls to be able to allocate Dr. Margarit, one for the secretary, and another bigger one for Registrars and SHOs. You can imagine how we were, a bit overcrowded. Then the problem was that we didn't have chairs, only a bookcase from the Hospital for the wall of the Registrar's room. A few days later, some furniture arrived that Dr. Margarit had bought to have the minimum things to start work. When we had our area organised, the Liver Transplant Unit opened.

After years of working, the dream of Dr. Margarit had come true — the first thing, the involvement, and the full integration of his team of physicians. They became the people who were called "The A Team". A reference team with high prestige that some of them are still enjoying today.

One day, he came in very happy and told me, "Montse, it seems they will allocate us in other offices on the 9th floor." This was difficult, but in the end, everybody had their space, their table, and chair. He bought a computer for each doctor and everybody started to work.

I remember a quotation of the writer John Ruskin saying "things we believe or think have no importance, in the end the only thing that is important is the things we are doing." This was Dr. Margarit's philosophy, the most important thing is to do.

Every day I will always remember him being in his office in the afternoon, in front of his computer, eating a sandwich made by himself because he didn't want to lose any moment of his valuable time.

Also, I remember when he came to my office and, looking across to the secretary's window, told me, "Montse, you are a privileged person, you have Barcelona at your feet, the capital of the World."

He would put his hand on my shoulder on and off without talking, to give me strength so that I would be able to continue the duties he asked of me. He was demanding, and he knew sometimes he asked me a lot, but I couldn't say no because everything he did was for science and for his patients.

We went to and from the Hospital to the Corachan Clinic, from the Corachan Clinic to the Quiron Clinic with the packed motorbike, with the Ultrasound, computers, briefcase...

One Tuesday evening, after the clinic, he showed me his photos of the trip he did with his son Pau and, thanks to technology, he was able to show me the same week.

My phone rang at 8 one morning with him telling me, "Good morning, Montse, can you come in a minute?" And this depends on the tone of his voice! I thought, "My God, what has he been thinking about during the night, because when he told me good night, he said nothing?" On his table, on recycled paper he brought from his home, he had written notes I called "my homework".

He never had an agenda for his ideas. He bought me a mobile phone so I could get rid of my pager, but he never understood why this device didn't have a signal in the lift. He even tried to change the supplier, but finally accepted this and several times I heard him on my voice mail telling me "I know you are in the lift!"

Little by little and with much effort, strength, tenacity, and fight, he obtained almost all the things he proposed for the Unit. Only one thing has never been granted, in spite of him asking for it constantly — another secretary for the Unit to carry on Hepatobiliary Surgery — but always was dismissed the request.

I want to talk about his dedication to the patients. His main concern was to save their lives. When an anxious patient came to see him, after his assessment, they left his office and had their hopes pinned on him and a lot of people told me “it seems he can give me a solution for my problem.” There were a few times when things went wrong, because sometimes reality is hard and it would show on his face when I asked him about the operation. If he wasn’t able to do anything, he answered me with a sorrowful attitude, upset.

He had a soft spot and concern for children and teenagers. He knew they had a future and all their lives in front of them. He always fought to search which way was the best to help them. I will always remember his enthusiasm on preparing the 20$^{th}$ anniversary of the first infantile liver transplant. Everything I showed him for the party he agreed with, but he told me, “What else can we offer them? Will they enjoy it?” and at the end of the party, he gave me a big thank you… he was happy and satisfied.

I remember a time when we spent all day at a friend’s house near the town of Vic and we were talking about the future and work. Dr. Margarit was asked when he would retire and he answered, “I will retire when Montse will.” I now can’t get rid of those words; I have my retirement soon.

Dr. Margarit was a natural and approachable person and gave to all the professional people he worked with and to those who asked for his opinion or help, he gave all the tools, knowledge, and advice to do and to develop their duties. He never said no to anybody, he didn’t have medical or scientific secrets, always encouraged everybody to publish their work, to prepare a thesis, etc. and in spite of this, he suffered an outrage after his death, as somebody stole his personal laptop and film from his camera which were in his office in the hospital.

I don’t want you to feel bitter for this event. Dr. Margarit chose Medicine and then Surgery and a good friend of mine says Surgery is a speciality that does not allow for mistakes. It demands a diagnostic accuracy and an impeccable technique and perfection with the mystery to discover, create a form of treatment, and the excitement of a result as a consequence of his personal action.

Art, science, and emotion. We can’t ask for more because a human activity is gratifying when the result is the well-being and the extension of human life. Surgery for me is a wonderful activity.

I think Dr. Margarit was born and lived to travel to Itaca; this travel, as Lluis Llach says in his song, we take far away, always far away, and when we think we are arriving, we find several paths to take. I believe he always had Itaca in his heart.

I thank Dr. Margarit for all the things he taught me and gave me, and feel lucky to have worked with him almost all his professional life.

Thank you Carles.

Montse Amenós
Vall d’Hebron, Spain

### Remembering My Father

Mr. Carlos Margarit Creixell, recognized Doctor, great friend, the best mountain partner: My Father.

Everything that comes to my mind is thank you. Always an example to follow, Carlos based his life in helping all his patients, family, and friends. Under his shy personality, you could find an open spirit, a big heart, and a transparent, easy going, generous, and very dedicated character. It's true he was serious, because he wanted everyone to succeed. He wanted me and all his students to learn to their greatest potential, and he wanted his medical team to work hard. Not looking for power or money, don't be mistaken, his goal was to help his patients and his students, and to provide the best education for his children. He did not believe in handing out presents, he believed in work, study, and nature. He was very generous. He always brought the camera to film the others, and gave his oranges and chocolate to us when we reached the mountain summit. I saw my father helping one of my friends to use his ski-bindings. I saw a Chamonix guide thanking my father at the top of the Mont Blanc summit after he had paved the way for others due to the deep snow. Carlos was a real sportsman. He finished seven marathons. The New York City Marathon was his best personal time: 3h.04min. He loved to wake up before anyone else, quietly walking around and staring at the sunrise; one of his favorite moments to connect with me.

My father showed me what the mountain was, how to love it, and to respect it. Hear the silence, enjoy the sunset and sunrise, and count the fire-stars in a vi-vac night. I remember my first summit with skis when I was 13 years old. La Tossa d' Alp (2547m) has always been Carlos' playground. After 15 years of intense mountain activity, it is impossible to count how many nights we spent under the sky, the countless slopes we skied down, and summits we had been on the top of. All these experiences gave him the energy to work hard and arrive at where he did with Medicine.

*The mountain is a place of connection:*

*A place where you feel part of nature,*
*A place where there is no hierarchy,*
*A place where respect and union between humans is necessary,*
*A place where you notice what's important in life and what is not,*
*A place where nothing can be hidden,*
*A place where you realize about LOVE, especially love to the others,*
*A place to remember,*
*A place to pray.*

I have to thank my father for showing me the way, the way to connect…

The mountain: the link between the land and the sky has been and will always be our place to communicate.

Every moment of fulfillment, I will shout "great victory" as my father always did when we reached the summit; "Gran Victoria"!!!

All of us, Carol, Eva, Anna, Laura and me feel proud to be a part of you.

Pau Margarit

### Images of Dr. Margarit's Professional Career, Family and Friends

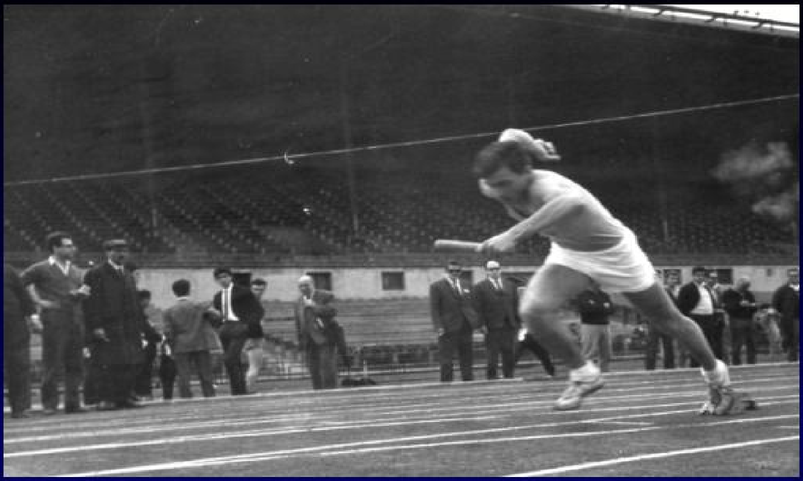

Carlos Margarit at 16 in a track and Field event while at the Jesuits High School in Sarria, Barcelona

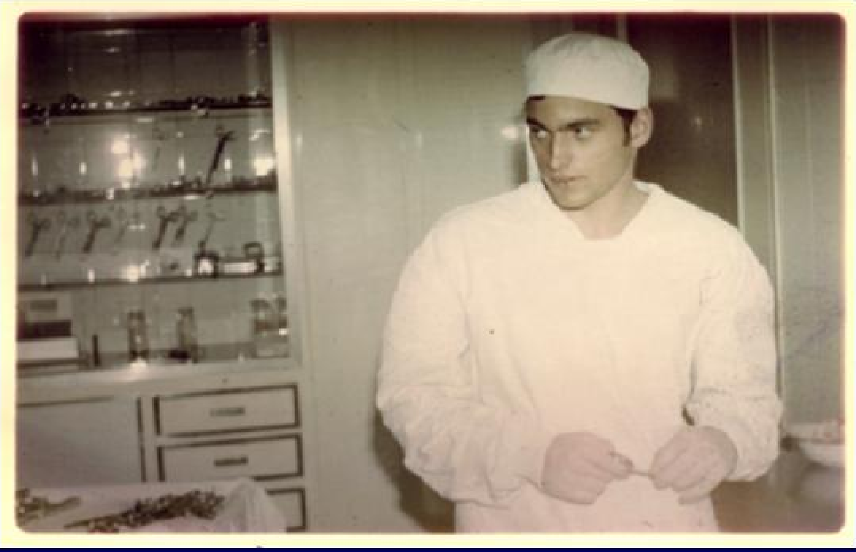

The young Dr. Margarit during surgery training.

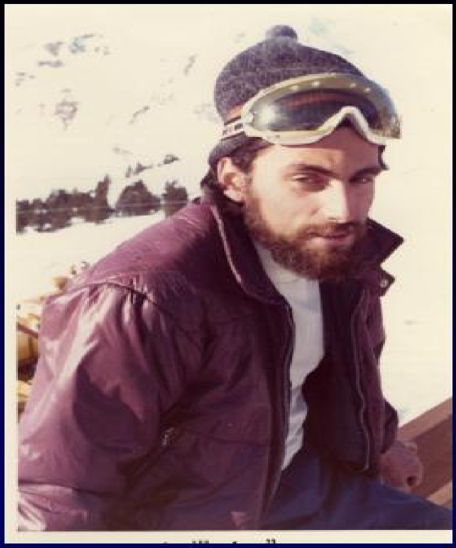

Carlos Margarit in his 20s in one of the many ski trips to village of Alp in the Spanish Pyrenees.

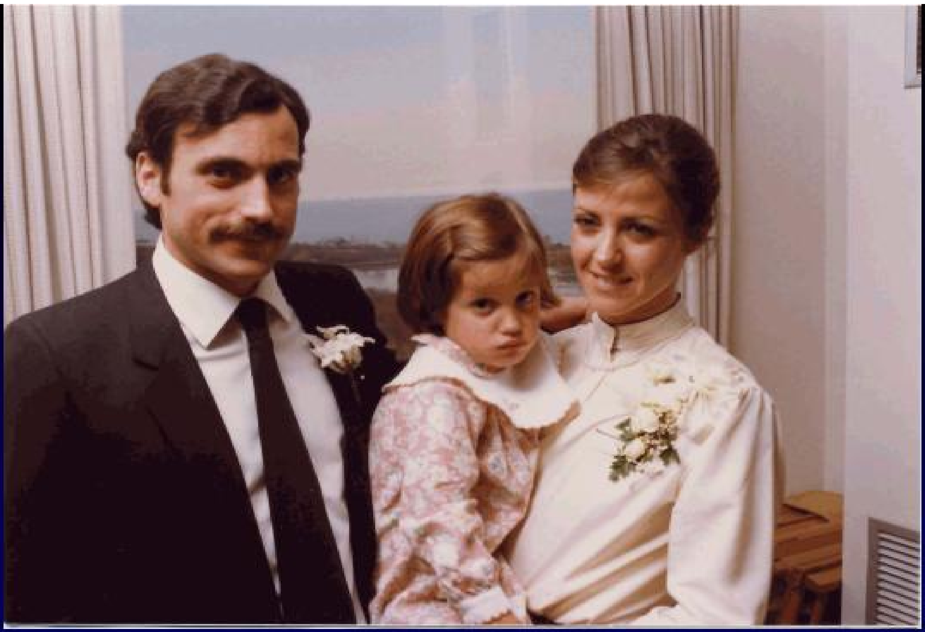

1983. Dr. Margarit with his first wife, Merce Serra and their daughter, Laura attending the wedding of Dr. Daniel Batlle in Chicago

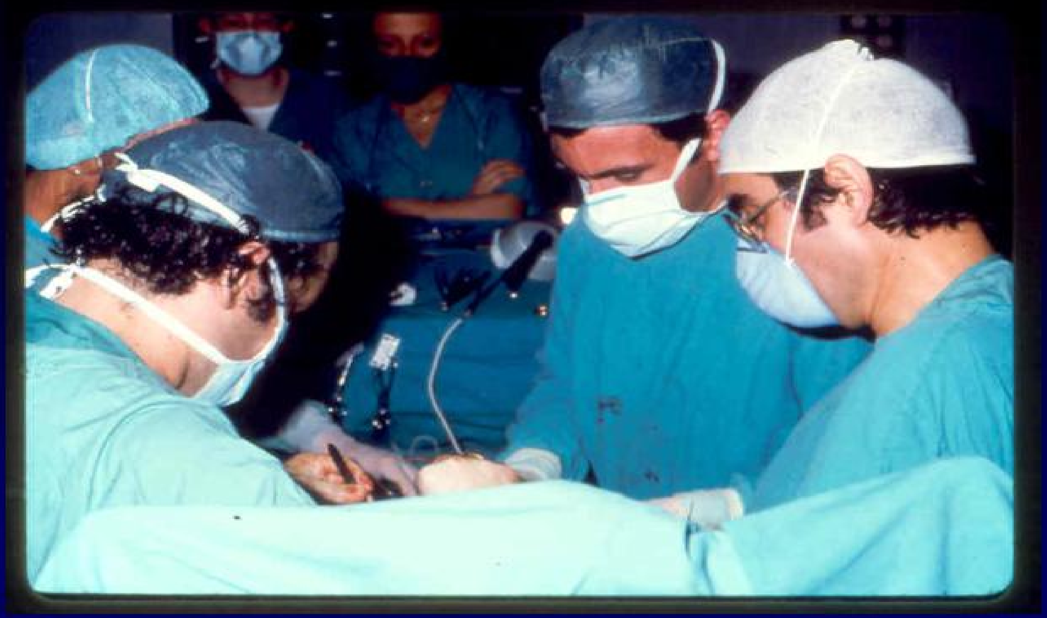

**1984 (First liver transplant in Spain) . Operating room in the Hospital of Bellvitge. Dr. Carlos Margarit (center) and collaborators**

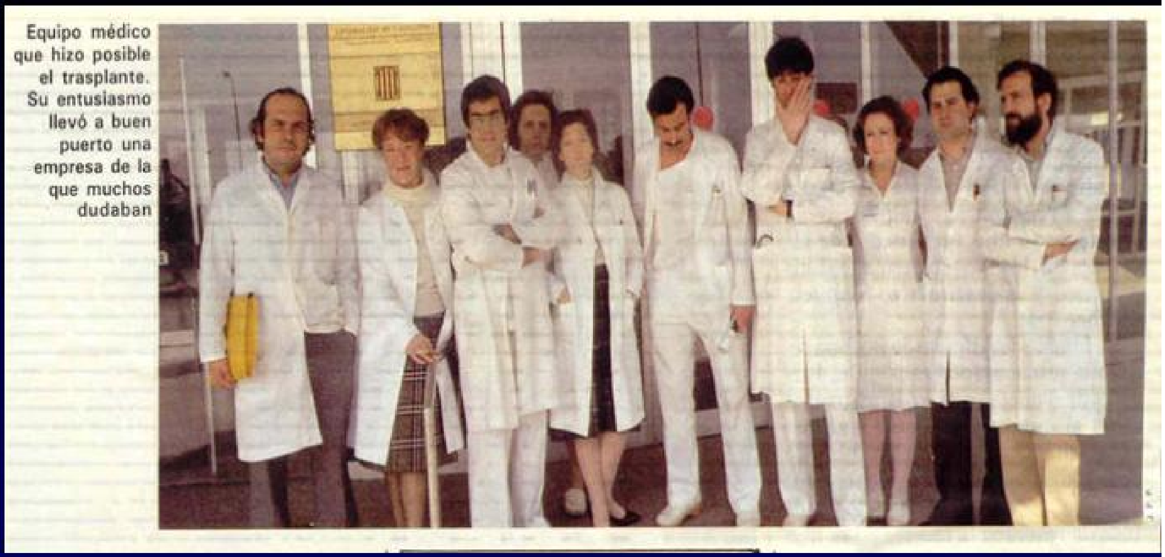

**Key members of the original liver transplant team from Hospital of Bellvitge in 1984 (Dr. Margarit looking down in the center).**

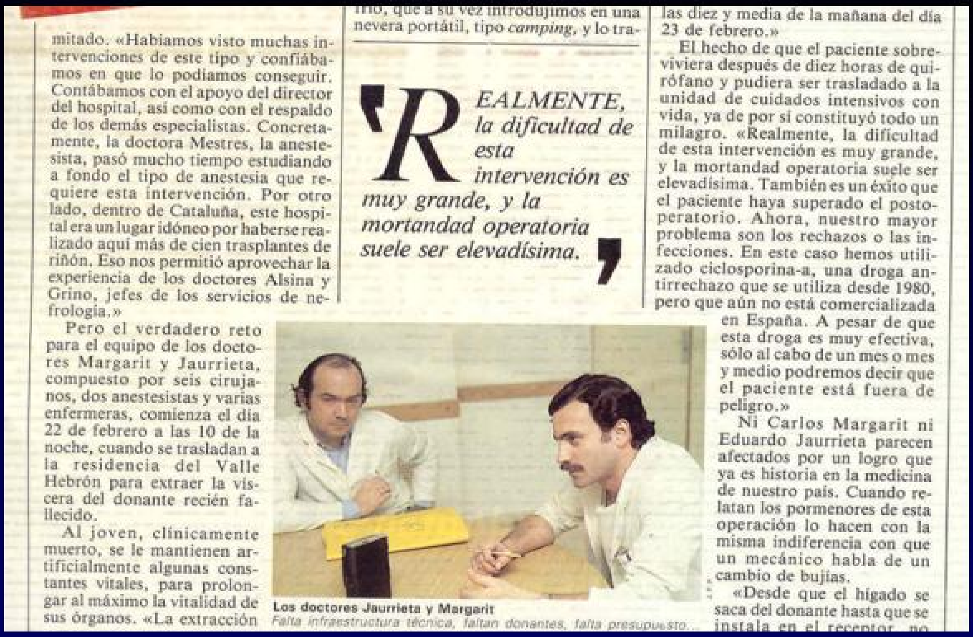

mitado. «Habiamos visto muchas intervenciones de este tipo y confiábamos en que lo podiamos conseguir. Contábamos con el apoyo del director del hospital, así como con el respaldo de los demás especialistas. Concretamente, la doctora Mestres, la anestesista, pasó mucho tiempo estudiando a fondo el tipo de anestesia que requiere esta intervención. Por otro lado, dentro de Cataluña, este hospital era un lugar idóneo por haberse realizado aquí más de cien trasplantes de riñón. Eso nos permitió aprovechar la experiencia de los doctores Alsina y Grino, jefes de los servicios de nefrología.»

Pero el verdadero reto para el equipo de los doctores Margarit y Jaurrieta, compuesto por seis cirujanos, dos anestesistas y varias enfermeras, comienza el día 22 de febrero a las 10 de la noche, cuando se trasladan a la residencia del Valle Hebrón para extraer la víscera del donante recién fallecido.

Al joven, clínicamente muerto, se le mantienen artificialmente algunas constantes vitales, para prolongar al máximo la vitalidad de sus órganos. «La extracción rrio, que a su vez introdujimos en una nevera portátil, tipo *camping*, y lo tra-

*'REALMENTE, la dificultad de esta intervención es muy grande, y la mortandad operatoria suele ser elevadísima.'*

Los doctores Jaurrieta y Margarit

*Falta infraestructura técnica, faltan donantes, falta presupuesto...*

las diez y media de la mañana del día 23 de febrero.»

El hecho de que el paciente sobreviviera después de diez horas de quirófano y pudiera ser trasladado a la unidad de cuidados intensivos con vida, ya de por sí constituyó todo un milagro. «Realmente, la dificultad de esta intervención es muy grande, y la mortandad operatoria suele ser elevadísima. También es un éxito que el paciente haya superado el postoperatorio. Ahora, nuestro mayor problema son los rechazos o las infecciones. En este caso hemos utilizado ciclosporina-a, una droga antirrechazo que se utiliza desde 1980, pero que aún no está comercializada en España. A pesar de que esta droga es muy efectiva, sólo al cabo de un mes o mes y medio podremos decir que el paciente está fuera de peligro.»

Ni Carlos Margarit ni Eduardo Jaurrieta parecen afectados por un logro que ya es historia en la medicina de nuestro país. Cuando relatan los pormenores de esta operación lo hacen con la misma indiferencia con que un mecánico habla de un cambio de bujías.

«Desde que el hígado se saca del donante hasta que se instala en el receptor, no

**1984. Media coverage of the first liver transplant in Spain by Dr. Margarit with a mustache and Dr. Jaurrieta.**

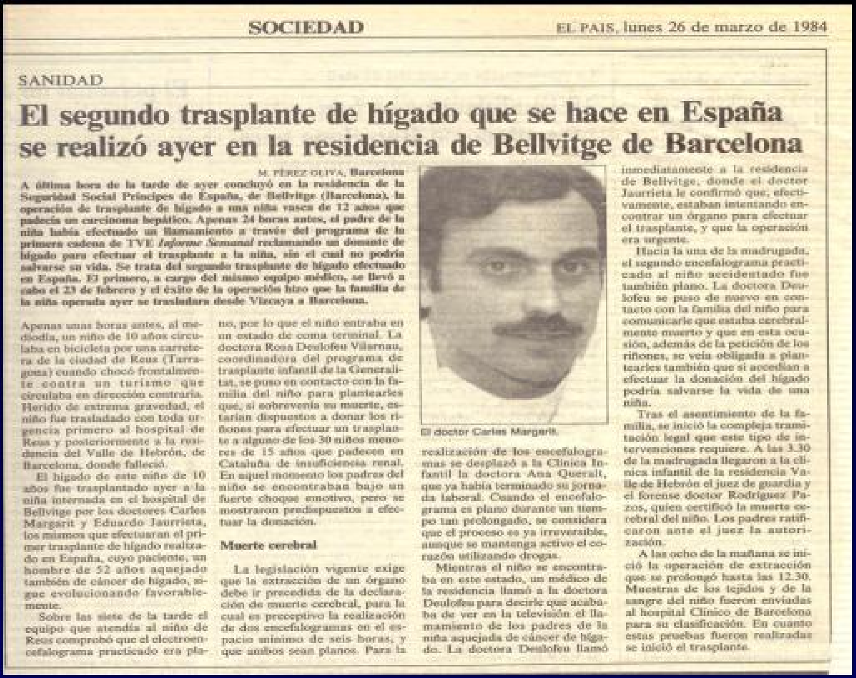

SOCIEDAD EL PAIS, lunes 26 de marzo de 1984

SANIDAD

### El segundo trasplante de hígado que se hace en España se realizó ayer en la residencia de Bellvitge de Barcelona

M. PÉREZ OLIVA, **Barcelona**

**A última hora de la tarde de ayer concluyó en la residencia de la Seguridad Social Príncipes de España, de Bellvitge (Barcelona), la operación de trasplante de hígado a una niña vasca de 12 años que padecía un carcinoma hepático. Apenas 24 horas antes, el padre de la niña había efectuado un llamamiento a través del programa de la primera cadena de TVE *Informe Semanal* reclamando un donante de hígado para efectuar el trasplante a la niña, sin el cual no podría salvarse su vida. Se trata del segundo trasplante de hígado efectuado en España. El primero, a cargo del mismo equipo médico, se llevó a cabo el 23 de febrero y el éxito de la operación hizo que la familia de la niña operada ayer se trasladara desde Vizcaya a Barcelona.**

Apenas unas horas antes, al mediodía, un niño de 10 años circulaba en bicicleta por una carretera de la ciudad de Reus (Tarragona) cuando chocó frontalmente contra un turismo que circulaba en dirección contraria. Herido de extrema gravedad, el niño fue trasladado con toda urgencia primero al hospital de Reus y posteriormente a la residencia del Valle de Hebrón, de Barcelona, donde falleció.

El hígado de este niño de 10 años fue trasplantado ayer a la niña internada en el hospital de Bellvitge por los doctores Carles Margarit y Eduardo Jaurrieta, los mismos que efectuaran el primer trasplante de hígado realizado en España, cuyo paciente, un hombre de 52 años aquejado también de cáncer de hígado, sigue evolucionando favorablemente.

Sobre las siete de la tarde el equipo que atendía al niño de Reus comprobó que el electroencefalograma practicado era plano, por lo que el niño entraba en un estado de coma terminal. La doctora Rosa Deulofeu Vilarnau, coordinadora del programa de trasplante infantil de la Generalitat, se puso en contacto con la familia del niño para plantearles que, si sobrevenía su muerte, estarían dispuestos a donar los riñones para efectuar un trasplante a alguno de los 30 niños menores de 15 años que padecen en Cataluña de insuficiencia renal. En aquel momento los padres del niño se encontraban bajo un fuerte choque emotivo, pero se mostraron predispuestos a efectuar la donación.

**Muerte cerebral**

La legislación vigente exige que la extracción de un órgano debe ir precedida de la declaración de muerte cerebral, para la cual es preceptivo la realización de dos encefalogramas en el espacio mínimo de seis horas, y que ambos sean planos. Para la realización de los encefalogramas se desplazó a la Clínica Infantil la doctora Ana Queralt, que ya había terminado su jornada laboral. Cuando el encefalograma es plano durante un tiempo tan prolongado, se considera que el proceso es ya irreversible, aunque se mantenga activo el corazón utilizando drogas.

Mientras el niño se encontraba en este estado, un médico de la residencia llamó a la doctora Deulofeu para decirle que acababa de ver en la televisión el llamamiento de los padres de la niña aquejada de cáncer de hígado. La doctora Deulofeu llamó inmediatamente a la residencia de Bellvitge, donde el doctor Jaurrieta le confirmó que, efectivamente, estaban intentando encontrar un órgano para efectuar el trasplante, y que la operación era urgente.

Hacia la una de la madrugada, el segundo encefalograma practicado al niño accidentado fue también plano. La doctora Deulofeu se puso de nuevo en contacto con la familia del niño para comunicarle que estaba cerebralmente muerto y que en esta ocasión, además de la petición de los riñones, se veía obligada a plantearles también que si accedían a efectuar la donación del hígado podría salvarse la vida de una niña.

Tras el asentimiento de la familia, se inició la compleja tramitación legal que este tipo de intervenciones requiere. A las 3.30 de la madrugada llegaron a la clínica infantil de la residencia Valle de Hebrón el juez de guardia y el forense doctor Rodríguez Pazos, quien certificó la muerte cerebral del niño. Los padres ratificaron ante el juez la autorización.

A las ocho de la mañana se inició la operación de extracción que se prolongó hasta las 12.30. Muestras de los tejidos y de la sangre del niño fueron enviadas al hospital Clínico de Barcelona para su clasificación. En cuanto estas pruebas fueron realizadas se inició el trasplante.

El doctor Carles Margarit.

**1984. Media coverage of the second liver transplant in Spain.**

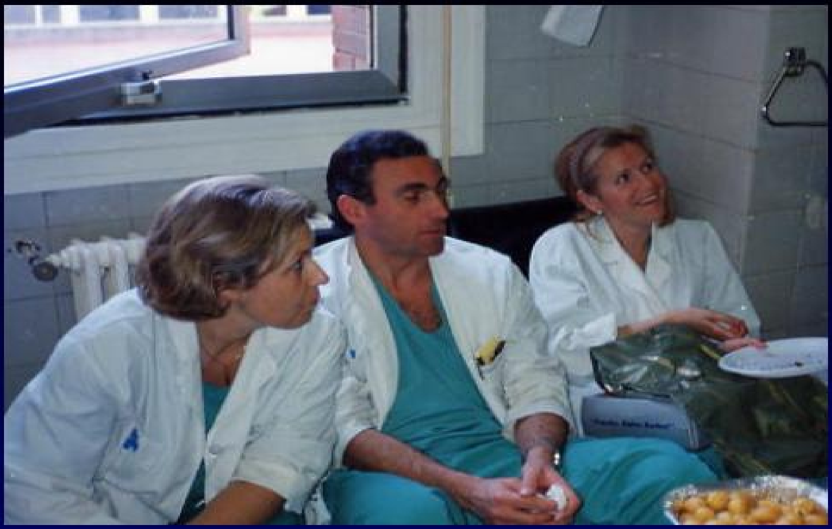

**1990. Dr. Margarit relaxing after surgery with two collaborators from the Hospital of Vall d' Hebron.**

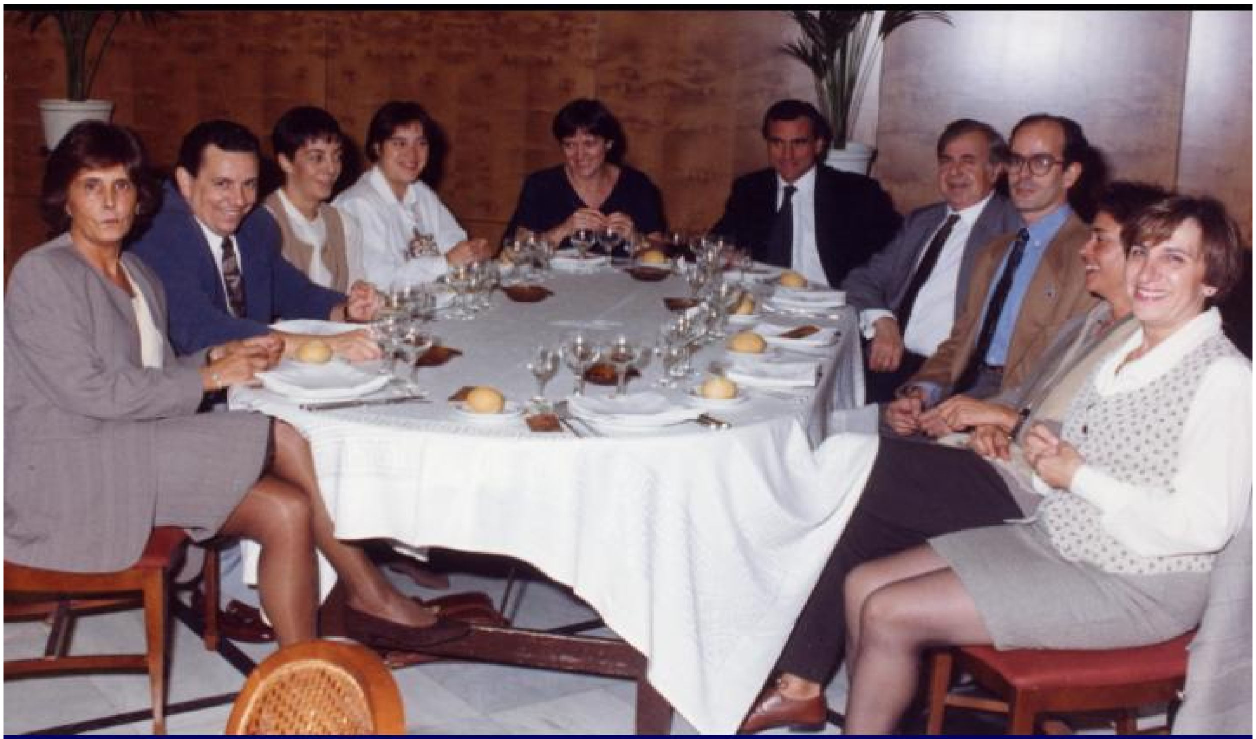

**1992. Celebrating the hundredth liver transplant in Hospital of Vall d' Hebron.**

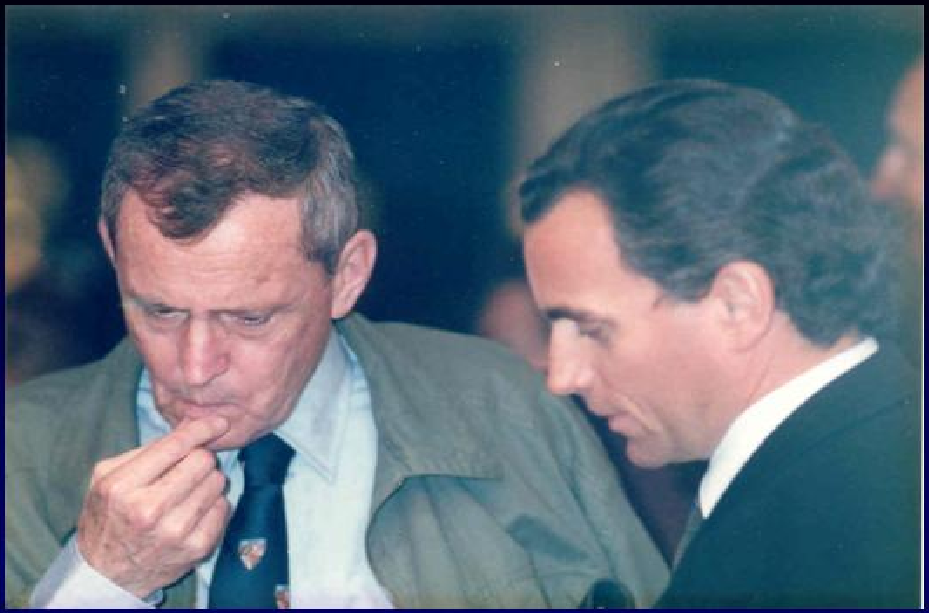

Dr. Carlos Margarit and Dr. Tomas Starzl

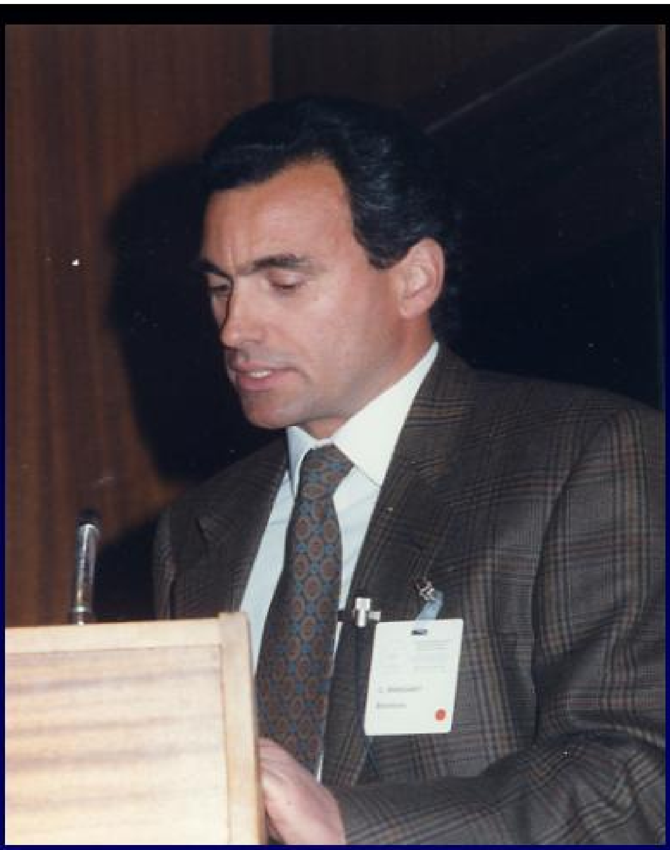

1992. Dr. Margarit giving a lecture.

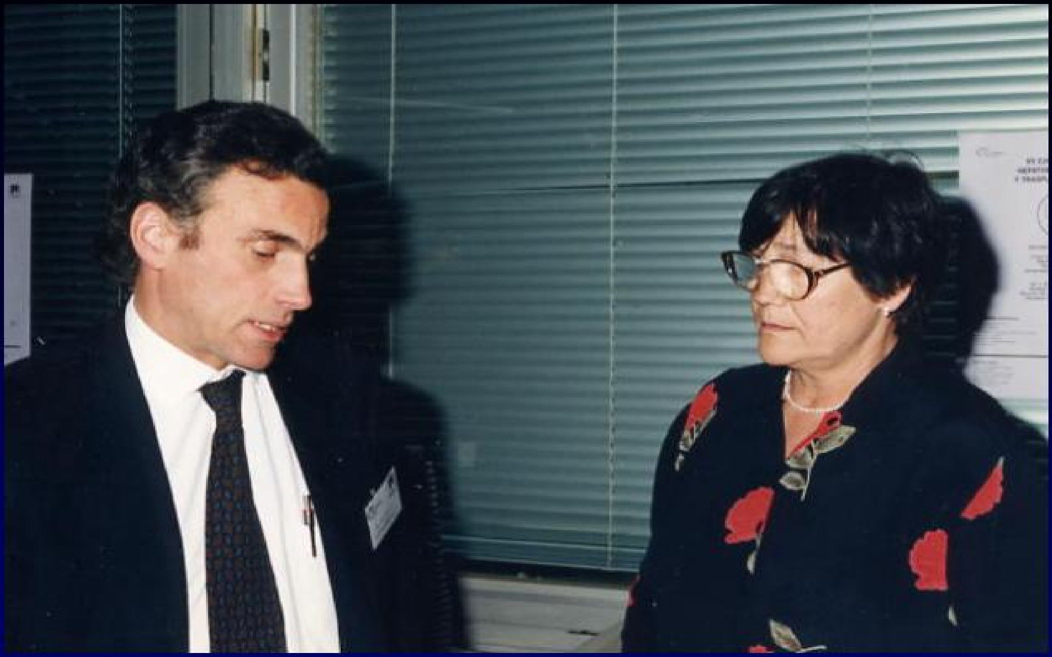

1992. Dr. Margarit with Montse Amenos his assistant of 20 years.

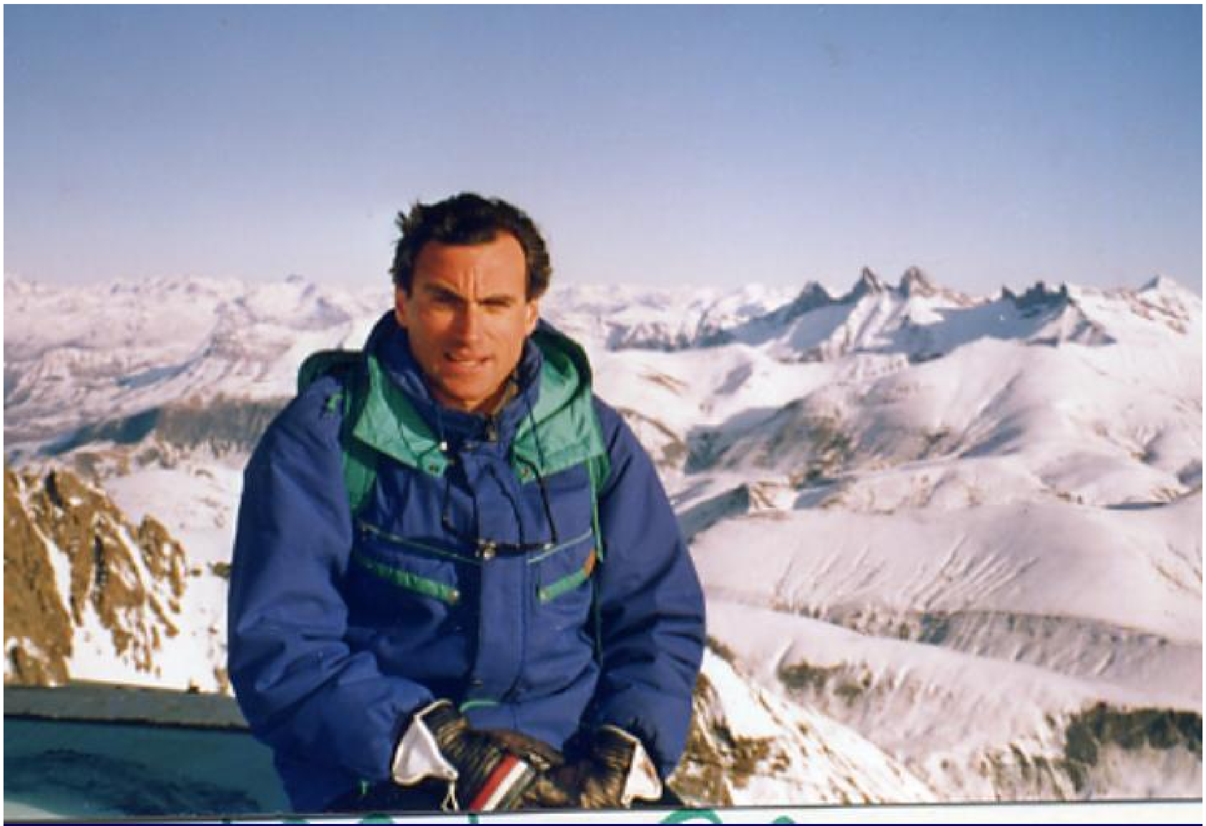

1993. Dr. Margarit at the top of the mountains, his favorite place.

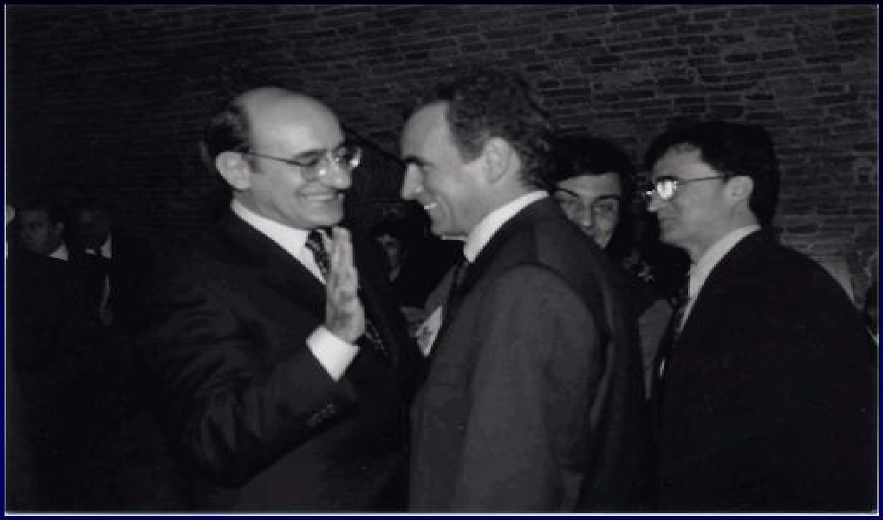

1997. Dr. Margarit at his wedding to Lluisa Colomer, being congratulated by Daniel Batlle. In the background are his two brothers, Ricardo and Jordi.

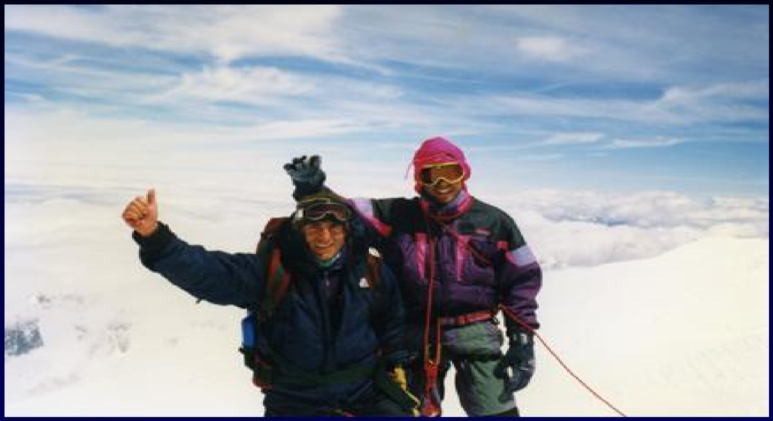

With his son Pau Margarit on top of Mont Blanc in one of his many ski trips

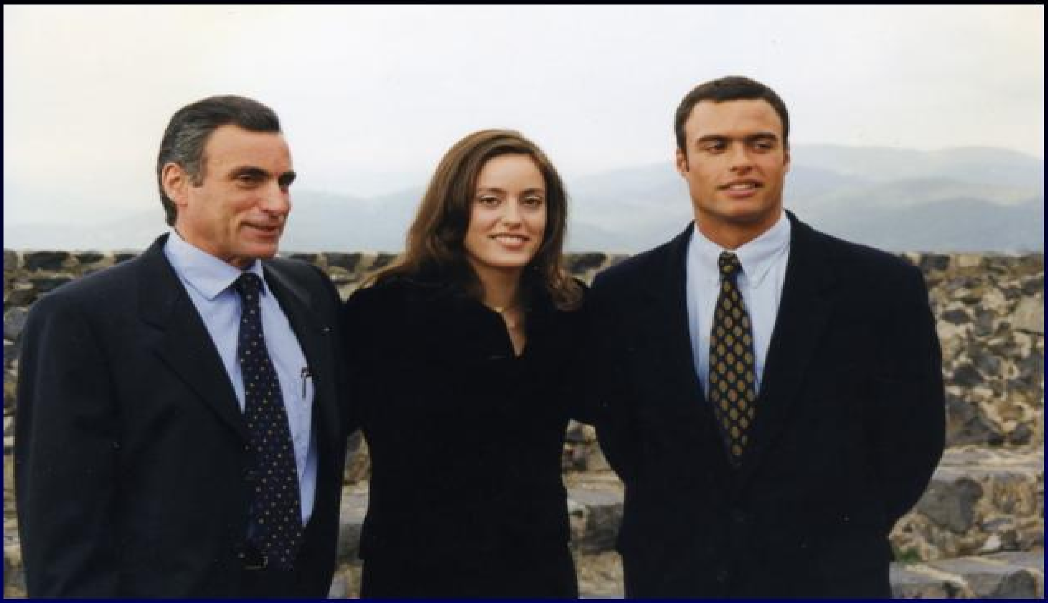

**1997. Dr. Margarit with his son and daughter, Pau and Laura**

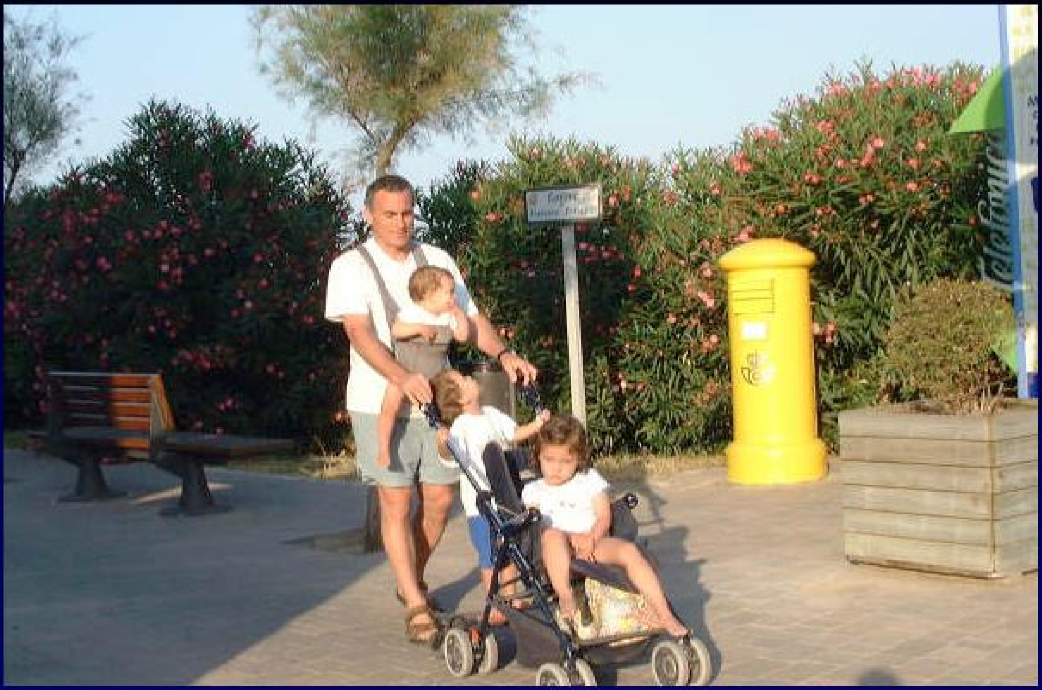

**Dr. Margarit with his three youngest daughters, Ana, Eva and Carol**

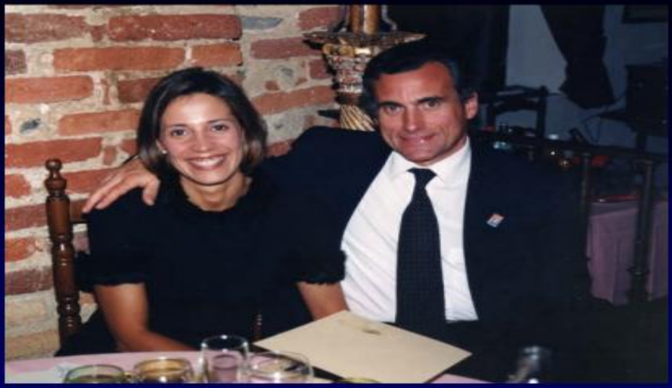

Dr. Margarit with his wife Lluisa Colomer

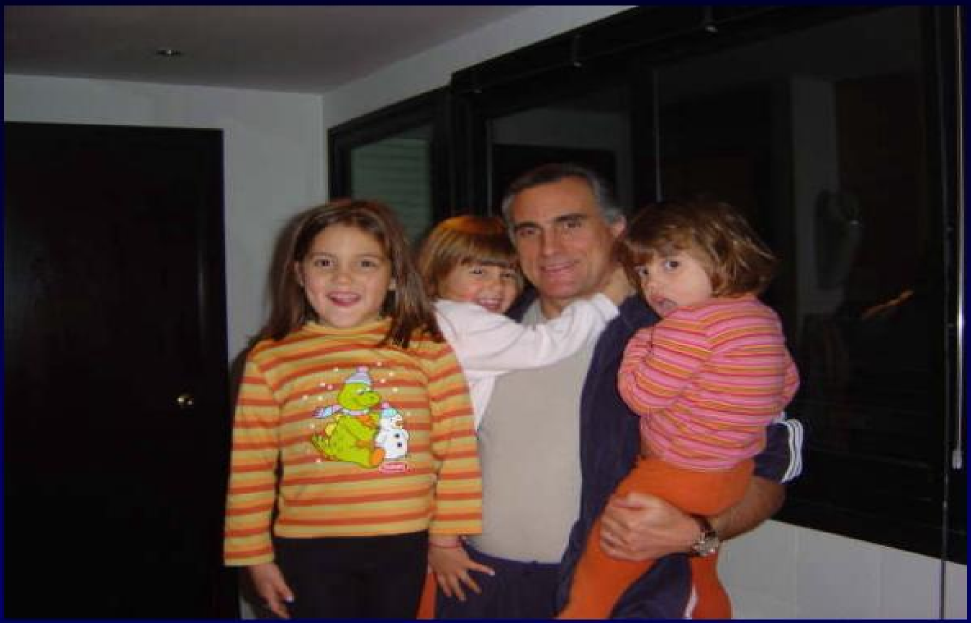

2005, the latest picture of Dr. Margarit with his three youngest daughters, Ana, Eva, and Carol

**This article should be cited as follows:**

Batlle, D. Carlos Margarit-In Memoriam, 1950-2005. (2006). *TheScientificWorldJOURNAL* **6**, 318–352. DOI 10.1100/tsw.2006.54.
